# Analysis of steroid hormones and their conjugated forms in water and urine by on-line solid-phase extraction coupled to liquid chromatography tandem mass spectrometry

**DOI:** 10.1186/s13065-016-0174-z

**Published:** 2016-05-06

**Authors:** A. C. Naldi, P. B. Fayad, M. Prévost, S. Sauvé

**Affiliations:** Department of Chemistry, Université de Montréal, Montreal, QC Canada; Department of Civil, Geological and Mining Engineering, Polytechnique Montréal, Montreal, QC Canada

**Keywords:** Conjugated steroid hormones, Solid phase extraction (SPE), Liquid chromatography tandem mass spectrometry (LC–MS/MS), Wastewater, River water, Urine, Estrogens

## Abstract

**Background:**

In recent years, endocrine disrupting compounds (EDCs) have been found in rivers that receive significant inputs of wastewater. Among EDCs, natural and synthetic steroid hormones are recognized for their potential to mimic or interfere with normal hormonal functions (development, growth and reproduction), even at ultratrace levels (ng L^−1^). Although conjugated hormones are less active than free hormones, they can be cleaved and release the unconjugated estrogens through microbial processes before or during the treatment of wastewater. Due to the need to identify and quantify these compounds, a new fully automated method was developed for the simultaneous determination of the two forms of several steroid hormones (free and conjugated) in different water matrixes and in urine.

**Results:**

The method is based on online solid phase extraction coupled with liquid chromatography and tandem mass spectrometry (SPE–LC–MS/MS). Several parameters were assessed in order to optimize the efficiency of the method, such as the type and flow rate of the mobile phase, the various SPE columns, chromatography as well as different sources and ionization modes for MS. The method demonstrated good linearity (R^2^ > 0.993) and precision with a coefficient of variance of less than 10 %. The quantification limits vary from a minimum of 3–15 ng L^−1^ for an injection volume of 1 and 5 mL, respectively, with the recovery values of the compounds varying from 72 to 117 %.

**Conclusion:**

The suggested method has been validated and successfully applied for the simultaneous analysis of several steroid hormones in different water matrixes and in urine.

**Electronic supplementary material:**

The online version of this article (doi:10.1186/s13065-016-0174-z) contains supplementary material, which is available to authorized users.

## Background

In the past decades, endocrine disrupting compounds (EDCs) have been observed in rivers that receive significant inputs of wastewater effluents. EDCs are chemicals with the potential to cause negative effects on the hormonal functions of humans and other animals with potentially harmful consequences, such as decreased fertility, development and growth problems in humans and hermaphroditism and feminization in animals [1, 2]. Among the large number of chemicals potentially responsible for endocrine disruption in wildlife, natural and synthetic estrogenic hormones have been considered as a matter of concern by scientists, water quality regulators and the general public [3]. Estrogens are known EDCs at the sub ng L^−1^ level [3, 4], while most of the other chemicals having an estrogenic effect are usually biologically active around the mg L^−1^ level [5–7].

Humans produce and excrete large quantities of endogenous estrogenic hormones. These natural hormones are excreted as sulfate or glucuronide conjugates mainly in urine [8, 9]. Synthetic estrogens are also of great interest due to their high estrogenic potency and the extent of their use. They have been used not only as contraceptives, but also for therapeutic purposes, in the management of hormone replacement therapy for menopausal women or in the treatment of various cancers, such as prostatic and breast cancer [2].

The contamination of the environment by estrogens can take place through the application of biosolids from municipal WWTP (wastewater treatment plant) on agricultural fields. However, the main pathway is usually through wastewater effluents, which after incomplete removal of these compounds in the municipal WWTP, are released into the receiving waters [10, 11].

Although the conjugated estrogens have been recognized to have a lower biologic activity than free (non-conjugated) estrogens, they can be cleaved to free estrogens. The presence of free estrogens in WWTP effluents and rivers [3, 10–15] indicated that estrogen metabolites could be converted back into active form before being released into the rivers. The cleavage of conjugated to free estrogens in the environment has not yet been well documented. Among the different hypotheses microbial processes before or during sewage treatment have been the most accepted hypothesis [16, 17]. *Escherichia coli* is known to be able to synthesize large amounts of the b-glucuronidase enzymes [18], and this has been suggested as the most probable mechanism responsible for the transformation.

Accurate detection and quantification of free and conjugated estrogens in rivers and wastewater is difficult to perform. The complexity of these matrices, the need to concentrate the samples due to the low concentration of the compounds, and the importance of sample integrity to avoid compound degradation all need to be considered. In previous works, estrogens and their conjugates were qualitatively and quantitatively determined by radioimmunoassay technique [12] or even by more sensitive and selective techniques, such as gas chromatography/mass spectrometry (GC–MS) [19, 20], or solid phase extraction (SPE) followed by liquid chromatography and tandem mass spectrometry, offline SPE–LC–MS/MS [14, 15].

SPE–LC–MS/MS seems to be the most promising currently available analytical technique to perform the detection and quantification of estrogens, since analytical methodologies based on radioimmunoassay techniques [21, 22] might overestimate estrogen concentrations and the GC techniques can be time-consuming and labor-intensive, often requiring derivatization and enzymatic hydrolysis prior to analysis [22, 23].

Immunoassays were extensively applied in the field of steroid determination in biological matrices. They have been replaced because of the problem with the cross-reactivity of various forms of common conjugates to the antibody. Immunoassays also require long preparation times, have limited dynamic range, and only allow the analysis of only one analyte at a time and cannot provide structural validation of the analyte [24].

Despite high resolution, lower operation cost and reduced solvent consumption, GC are less commonly used for the analysis of steroids than LC, mainly due to the difficulty of sample preparation, as derivatization should be applied in all studies with GC–MS determination [25].

Off-line SPE is one of the most common methods used to concentrate analytes and remove matrix interferences to achieve the desired levels of analytical sensitivity [26, 27]. However, this process can be labor-intensive, often requiring many steps and the need for large sample volume. The development of on-line SPE methods, by coupling SPE to the LC system using a column-switching technique could be an advantageous. It eliminates several required steps (namely evaporation and reconstitution), reduces sample manipulation as well as preparation time in comparison to off-line SPE. The automation of on-line SPE results in better repeatability and reproducibility, which helps to improve the quality of the reported analytical data. Higher sample throughput increases the number of samples that can be analyzed in a single day. In addition, smaller sample volume and solvent requirements reduce the costs of consumables and the environmental footprint [28, 29].

Although automated on-line methods have clearer advantages over off-line SPE [30], the development of on-line methods can be challenging. The transfer of off-line methods to on-line mode may lead to an incompatibility between SPE sorbents and analytical columns, adjustment of mobile phases, pH incompatibility and peak broadening [31]. In addition, to achieve comparable pre-concentration factors to off-line SPE, it is possible to increase the on-line injection volumes. In this case, breakthrough volume estimation is necessary to guarantee that the compounds are fully retained during the loading of the SPE the column and that there are no losses of analytes [32, 33].

In this study, a fully automated on-line solid-phase extraction–liquid chromatography–mass spectroscopy detection (SPE–LC–MS/MS) is presented. It allows for the simultaneous detection of both estrogens forms (conjugated and free) in urine and water samples. In order to confirm the presence (or absence) of conjugated and free estrogens and the applicability of the method in urine and real environmental samples, the determination of the selected conjugated and free estrogens hormones at low-nanogram per liter levels was done. Urine samples from pregnant women and women of reproductive age were analyzed. Wastewater and effluent samples from the Repentigny wastewater treatment facility (north-east of Montreal, QC, Canada) and river samples from four different locations: Thousand Islands River, Saint Lawrence River (at Delson), Des Prairies River and Saint Lawrence River (at Repentigny), all in the province of Quebec, Canada were analyzed. The method has been validated by evaluating the linear range, accuracy and precision (intra-day and inter-day).

## Experimental

### Standards and reagents

**Fig. 1:**
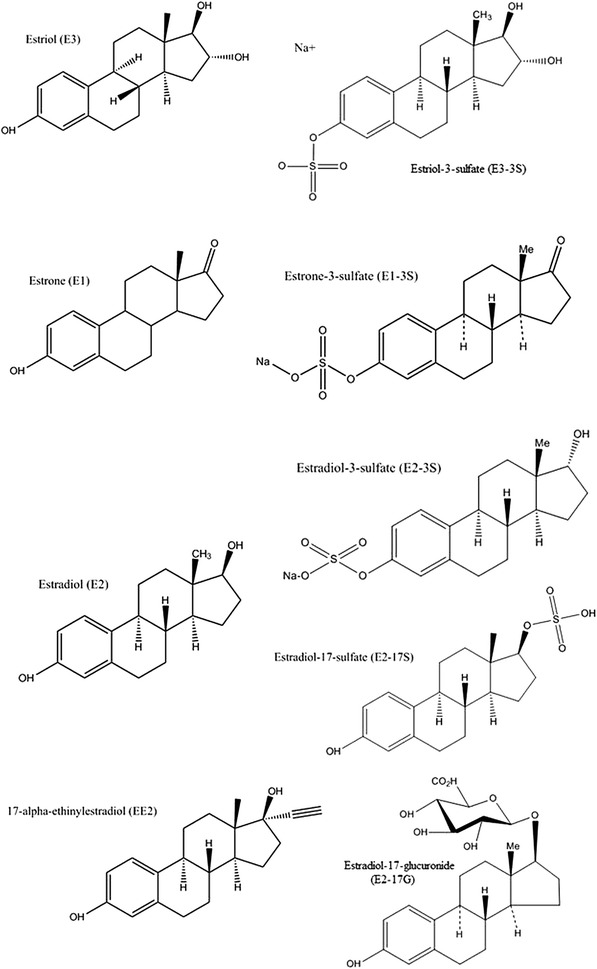
Chemical structures of target free and conjugated estrogens (drawn using ChemBioDra Ultra 14.0)

Conjugated estrogens standards (estriol-3-sulfate (E3-3S), estradiol-3-sulfate (E2-3S), estrone-3-sulfate (E1-3S), estradiol-17-sulfate (E2-17S), estradiol-17-glucoronide (E2-17G)), and the internal standard [estradiol-d4-3-sulfate (E2-d4-3S)] were obtained from Steraloids Inc. (Newport, RI, USA). Free estrogens standards [estriol (E3), estrone (E1), estradiol (E2) and 17-alpha-ethinylestradiol (EE2)], and the internal standard [^13^C_6_]-estradiol were purchased from Sigma–Aldrich (St. Louis, MO, USA). The chemical structures of the estrogens studied are shown in Fig. 1. Other solvents and reagents (trace analysis grade), methanol (MeOH), ammonium hydroxide (NH_4_OH) and HPLC-grade water were purchased from Fisher Scientific Inc. (Whitby, ON, Canada). Individual stock solutions for all compounds were prepared by dissolving accurately-weighed samples in HPLC-grade methanol to obtain a final concentration of 1000 µg mL^−1^. These solutions were kept at −20 °C. Standard solutions containing all compounds were mixed and diluted with methanol. Standard working solutions of all compounds and calibration concentrations were prepared daily by serial dilution with HPLC-grade water (95 % H_2_O, 5 % MeOH maximum v/v).

### Instrumental conditions

**Fig. 2:**
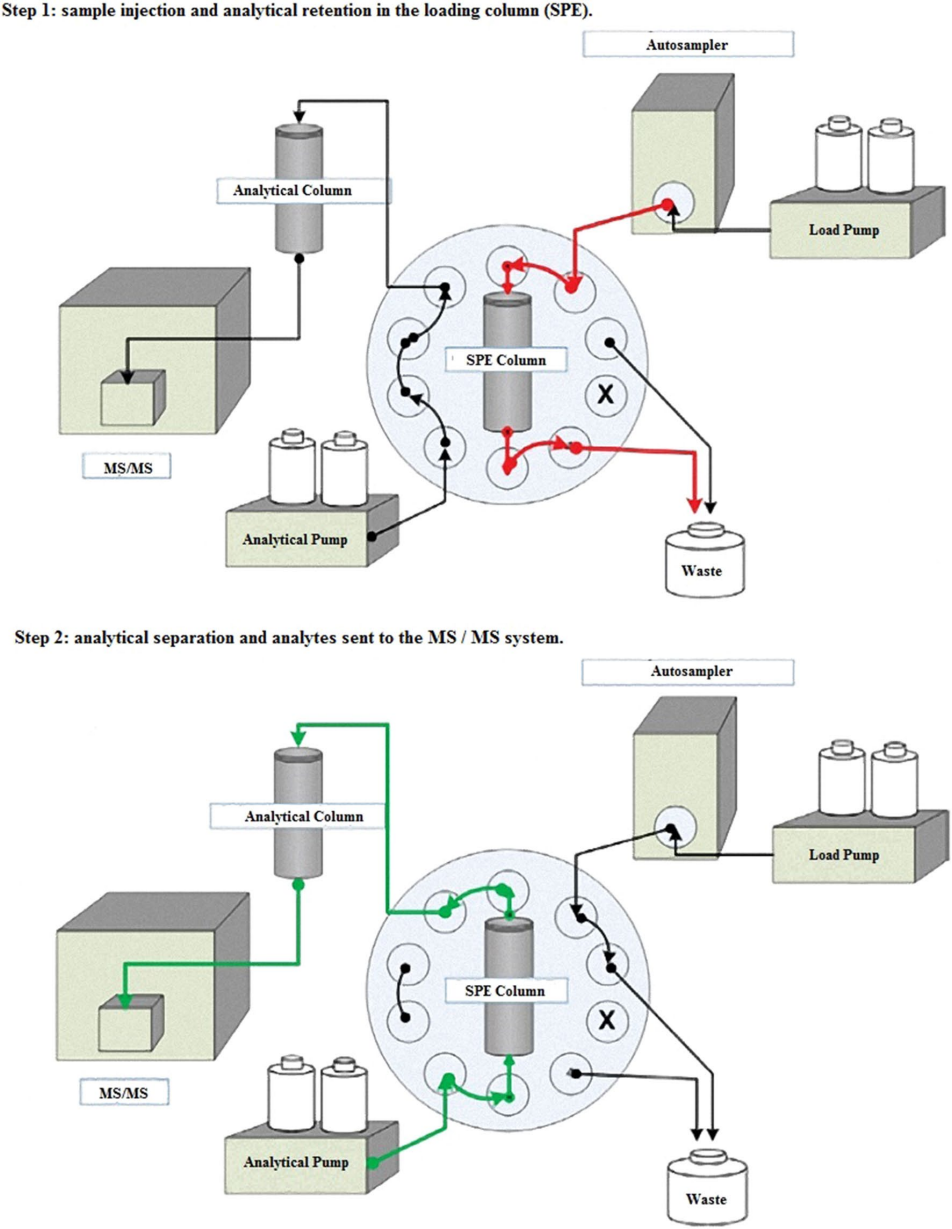
The EQuan™ system (column-switching technique) schema used in this experiment

Sample pre-concentration and separation were performed using the EQuan™ system (Thermo Fisher Scientific, Waltham, MA, USA) combined with detection using a Quantum Ultra AM tandem triple quadrupole mass spectrometer fitted with an HESI source. The EQuan™ system was based on a column-switching technique as shown in Fig. 2. The instrument was operated in negative ionization mode for the selected compounds of interest and was directly coupled to the HPLC system. A column switching technique was used to perform the on-line SPE–LC–MS/MS analysis. Sample analysis was performed in the selected reaction monitoring mode (SRM). System control and data acquisition were performed using the Analyst Xcalibur software (rev. 2.0 SP2, Thermo Fisher Scientific, USA).

### On-line solid phase extraction

The column switching system combines a six-port and a ten-port valve (VICI^®^ Valco Instruments Co. Inc., Houston, TX, USA). This technique allowed the injection and pre-concentration of samples using a high-pressure pump, a low-pressure pump, a load column and an analytical column.

The samples were injected using a HTC thermopal autosampler (CTC analytics AG, Zwingen, Switzerland). Two different sample volumes were injected in the system (1 and 5 mL). In the first case, the instrument was programmed to draw 1.2 mL of the sample from the vial and inject it in the 1 mL injection loop. In the second case, it was programmed to draw three times 2.5 mL (total of 7.5 mL) of the sample from the vial and inject it in the 5 mL injection loop. The excess of sample was injected to guarantee that the loop was completely filled and to reduce the sample dilution effect inside the loop during the injection process [32].

The samples were then pre-concentrated on the loading column (BetaBasic 20 × 2.1 mm, 5 µm particle size in DASH, Thermo Fisher Scientific, USA) with 60 % of solvent A (0.1 % NH_4_OH, H_2_O) and 40 % of solvent B (0.1 % NH_4_OH, MeOH) using the load pump (low-pressure quartenary pump Accela 600, from Thermo Fisher Scientific, USA) at a flow rate of 1000 μL min^−1^. The valve position was then switched to allow the bound material to be eluted from the extraction cartridge in back flush mode directly onto the analytical column (Betabasic 18, 100 × 2.1 mm, 3.0 μm particle size, Thermo Fisher Scientific, USA) coupled with a guard column using the same packing material (10 × 2.1 mm/3.0 μm, Thermo Fisher Scientific, USA). A high-pressure quaternary pump Accela 1250, from Thermo Fisher Scientific, USA was used for liquid chromatography (analytical pump).

Optimization of the on-line sample pre-concentration was done by a series of tests to study the behaviour of the system to variations of key parameters such as column type, sample load flow rate, volume of the load column wash and organic solvent content of the load column wash.

### Chromatographic conditions

Once the analytes retained by the load column (SPE) were gradually eluted by back flushing and then introduced in the LC system (guard column and analytical column), where chromatographic separation took place. The analytical pump gradient was composed of solvent A: 0.1 % NH_4_OH, H_2_O and solvent B: 0.1 % NH_4_OH, MeOH. The gradient elution program is shown in Additional file 1 (for a 1.0 and 5.0 mL loop, respectively). Column temperature was set to 30 °C. Separated compounds were then introduced to the MS inlet for analysis.

All the operations were fully automated with a separation time of 10 min and a total run time of 20 min. To avoid sample cross contamination, the syringe and the injection valve were washed twice with 5 mL of a mix of ACN/iso-Propanol/MeOH (1/1/1; v/v/v) and H_2_O after each injection.

### Mass spectrometry

Optimization of the mass spectrometry (MS) was performed. Key parameters such as ionization source (HESI and APCI), ionization modes (negative and positive), spray voltage, sheath gas pressure, auxiliary gas pressure and capillary temperature were tested in order to achieve the highest possible sensitivity. The best conditions of ionization of analytes were obtained using heated electrospray ionization in negative mode (HESI-). Ion source parameters were optimized for each compound using the Quantum Tune application of Xcalibur software (rev. 2.0 SP2, Thermo Fisher Scientific, USA) which was also used to control the instrument and for data acquisition. Individual standard solutions (10 mg L^−1^) were infused with the syringe pump and mixed using a tee with the LC flow, mobile phase solvent A: 0.1 % NH_4_OH, H_2_O and solvent B: 0.1 % NH_4_OH, MeOH (50:50), (300 μL min^−1^), before being introduced into the HESI source. The full-scan mass spectra and the MS/MS spectra of the selected compounds were obtained for all analytes. The selected reaction-monitoring mode (SRM) was performed for the detection of the two most intense transitions at their respective m/z ratios. The most intense SRM transition (SRM#1) was selected for quantitation and the second most intense (SRM#2) was used for confirmation. SRM transitions, collision energy and skimmer offset were compound-dependent and appear in Table 1. The identification of analytes was confirmed by the LC retention time [34–36].

**Table 1:** Tandem mass spectrometry (MS/MS) optimized parameters for the analysis of selected estrogens hormones in negative (NI) ionization mode

Hormone	Ion	SRM#1	Collision energy (V)	SRM#2	Collision energy (V)	Tube lens (V)
E3-3S	367	287	38	80	33	−98
E2-17G	447	271	31	325	28	−94
E2-3S	351	271	37	145	48	−93
E1-3S	349	269	36	145	53	−90
E2-17S	351	97	41	80	42	−96
E2-d4-3S	355	275	40	–	–	−91
E1	269	145	41	159	41	−94
E2	271	145	47	183	44	−95
EE2	295	145	48	159	38	−100
E3	287	14	44	171	37	−98
13C6-E2	277	145	48	–	–	−101

For the compound E1-3S only one transition was used in water matrix as the second transition is not intense enough for the identification and quantification of this compound in the desired concentration range. The second transition for this compound showed satisfactory results only for concentrations of at least 200 ng L^−1^ and was used in urine samples.

A basic additive, ammonium hydroxide (NH_4_OH), was added to the mobile phase to improve dissociation of the phenol group and improve the sensitivity [37, 38].

### Breakthrough volume estimation

Breakthrough volume estimation experiments are usually done using the graphical extrapolation method [36]. However, they can also be done experimentally; optimizing the SPE loading speed and the sample volume that can be charged in the column without loss of analytes [39].

The breakthrough volume for the selected estrogens was established by injecting different sample volumes (1, 2, 5 and 10 mL) and comparing absolute areas and signal-to-noise values. Tests were done in duplicate, with triplicate samples each time. Samples were prepared daily at the same concentration (500 ng L^−1^) in HPLC water, using 1, 2, 5 and 10 mL loops. Results were analysed using linear regression to determine the maximum injection volume.

### Matrix effects study

Matrix effects are very important when developing a method, since they might affect reproducibility and accuracy [34, 35, 40–43]. Matrix effects were evaluated by comparing the results of spiked (50–200 ng L^−1^) HPLC water samples with those measured in tap water, river water and wastewater spiked with the same amounts of analytes. The absolute matrix effect was calculated as:

$${\text{Matrix Effect }}\left( \% \right)\; = \;\left( {{{{\text{C}}_{\text{matrix}} } \mathord{\left/ {\vphantom {{{\text{C}}_{\text{matrix}} } {{\text{C}}_{\text{HPLC}} }}} \right. \kern-0pt} {{\text{C}}_{\text{HPLC}} }}} \right) \times 100$$

where C_matrix_ = measured concentration in the tap water, river water and wastewater sample, C_HPLC_ = measured concentration in HPLC water.

A value of 100 % indicates that there is no absolute matrix effect. If the value is >100 %, there is a signal enhancement while a signal suppression is observed if the value is <100 %. These experiments were performed with five replicates.

### Method validation and calibration

The performance of the method was evaluated through estimation of the recovery, linearity, repeatability (intra-day precision), intermediate precision (inter-day precision), accuracy, limit of detection (LOD) and limit of quantification (LOQ).

The recovery for the online SPE method was evaluated at two different concentrations (500 and 1000 ng L^−1^, n = 5). The mean peak areas (20 and 40 µg L^−1^, n = 5) of the selected estrogens of a direct injection (25 µL) were compared with those of the on-line 1 mL volume injection. The same mass of analyte was injected in both cases [39].

Calibration curves were established in urine, HPLC-grade water, tap water, river water and wastewater in order to avoid the influence of matrix effects on linearity. At least five-point calibration curves were established for the analytes in aqueous samples (5–5000 ng L^−1^ injected in duplicate or triplicate). The calibration range was chosen based on the method analytical performance and the concentrations found for these compounds in the literature [1, 15, 23, 37, 44–47]. Quantification for all compounds was performed using a standard addition calibration with linear regression and isotopically-labelled internal standards between 0.25 and 1 μg L^−1^. Calibration curves were built with the response ratio (area of the analyte standard divided by area of the internal standard) as a function of the analyte concentration. A linear regression model was applied, with coefficients of determination (R^2^) greater than 0.993 for all analytes.

Accuracy was evaluated by comparing the results of spiked tap water, river water, wastewater and urine samples (50–200 ng L^−1^ for water samples and 500–5000 ng L^−1^ for urine samples) with the nominal spike concentration. The accuracy was calculated as:

$${\text{Accuracy }}\left( \% \right) \; = \;100 - \left[ {{{\left( {{\text{C}}_{\text{e}} {-}{\text{C}}_{\text{m}} } \right)} \mathord{\left/ {\vphantom {{\left( {{\text{C}}_{\text{e}} {-}{\text{C}}_{\text{m}} } \right)} {\left( {{\text{C}}_{\text{e}} } \right)}}} \right. \kern-0pt} {\left( {{\text{C}}_{\text{e}} } \right)}} \times 100} \right]$$

where C_m_ = measured concentration, C_e_ = expected concentration.

The method repeatability (intra-day precision) and reproducibility (inter-day precision) were evaluated from the analysis of replicates of urine, HPLC-grade water, tap water, river water and wastewater spiked with a standard mixture of the analytes between 50 and 200 ng L^−1^. The repeatability and reproducibility were defined as the relative standard deviation (%) of the response ratio.

Five samples (n = 5) were used to estimate repeatability while twelve samples (n = 12) were used to estimate reproducibility. Samples were prepared daily and analyzed in the analytical sequence.

Seven to ten samples (n = 7–10) were spiked with all the analytes of interest at a concentration from two to five times the estimated detection limit and carried through the analytical process and analyzed. The limit of detection (LOD) was determined by multiplying the appropriate statistical Student’s t-value (3.143 for seven replicates) by the standard deviations of the analyzed replicate samples. To be considered acceptable, the level of analyte in the sample must be above the determined LOD and not exceed ten times the LOD of the analyte in reagent [48].

Quantification limit (LOQ) was estimated from LOQ from the equation:

$${\text{LOQ}} \; = \;{\text{LOD}}\; \times \;3$$

Sample carryover was evaluated by injecting a series of blanks (n = 4) after a high concentration standard (2000 ng L^−1^) in every sequence.

$${\text{Carryover }}\left( \% \right) \; = \;{{{\text{C}}_{\text{blank}} } \mathord{\left/ {\vphantom {{{\text{C}}_{\text{blank}} } {{\text{C}}_{\text{standard}} }}} \right. \kern-0pt} {{\text{C}}_{\text{standard}} }} \times 100$$

where C_blank_ = concentration in the blank sample, C_standard_ = concentration of the 2000 ng L^−1^ spiked sample.

An appropriate retention time window for each analyte has been established in order to identify them in quality control sample (QC). Measurements of the actual retention time variation for each compound in standard solutions over time has also been obtained chromatograms of field –collected samples. The positive identification of the estrogens was confirmed by matching chromatographic retention times with those from spiked samples in HPLC water (analyte-free matrix). The suggested variation is plus or minus three times the standard deviation of the retention time for each compound for a series of injections [49]. In addition, at least two selected reaction monitoring (SRM) transitions were selected for each target compound and their relative intensities were compared. In accordance with the European Commission, Council Regulation (EEC), [50] the SRM transitions ratios were considered acceptable if the error was within ±50 % since their relative intensities were inferior to 10 %.

### Environmental samples/sample collection and preservation

Water samples from a variety of sources in the Montreal area, were collected.

Sewage and effluent samples were collected from the Repentigny wastewater treatment plant facility (WWTP). In the wastewater treatment plant in Lebel Island, the wastewater treatment involves physical and chemical processes, as well as a biological sludge process. This WWTP is part of the short list of plants in Quebec to produce its own biogas. The biogas is produced by the anaerobic digestion of the sludge and it is recovered for several uses, including heating the facility.

River water samples were collected in Saint-Lawrence River (near Delson and Repentigny), in the Des Prairies River and in the Milles Iles River. They were selected due to the documented discharges of urban and agricultural wastes [34, 41]. Drinking water samples were collected directly from the Université de Montréal’s tap water (Montreal’s aqueduct).

Urine samples were kindly obtained from six different women (three pregnant women and three women of reproductive age, between 15 and 40 years old). Pregnant women were in the third trimester of their pregnancy (between 28 and 40 weeks).

All samples were collected in clean glass bottles and then immediately transported to the laboratory. The samples were filtered using 1.2 mm glass fiber filters (Millipore, MA, USA) followed by 0.3 mm glass fiber membranes filters (Sterlitech Corporation, Kent, WA), stored in the dark at 4 °C and analyzed within 48 h. A previous study showed that this filtration step did not cause analyte losses [39]. Aliquots of 10–30 mL of the water and urine samples were transferred to volumetric flasks and spiked with the IS for a final concentration of 200–500 ng L^−1^. The samples were then transferred to 10 mL amber glass vials for on-line SPE–LC–MS/MS analysis.

## Results and discussion

### On-line trace enrichment

Three different SPE columns were tested: Hypersil Gold aQ. column, 20 × 2.1 mm, 12 μm, Thermo Fisher Scientific, USA; Hypercarb column, 20 × 2.1 mm, 7 μm, Thermo Fisher Scientific, USA and BetaBasic, 20 × 2.1 mm, 5 μm, in DASH, Thermo Fisher Scientific, USA (data not shown). The best recovery values were found using a BetaBasic (Table 2). Important on-line SPE parameters such as sample loading flow rate, wash volume and organic modifier in the wash volume were optimized to obtain optimal results in relation to system stability and run time using the BetaBasic.

**Table 2:** Recovery values in percentage for the selected estrogens using the SPE BetaBasic column in HPLC water samples

Estrogens	Recovery (%)
E3-3S	117
E2-17G	98
E2-17S	96
E1-3S	88
E2-3S	103
E3	95
E2	96
E1	94
EE2	72

Recovery values were calculated comparing off-line small injection method (25 μL) with online 1 mL injections (same mass of analyte injected) (C = 500 ng L^−1^, n = 5)

**Fig. 3:**
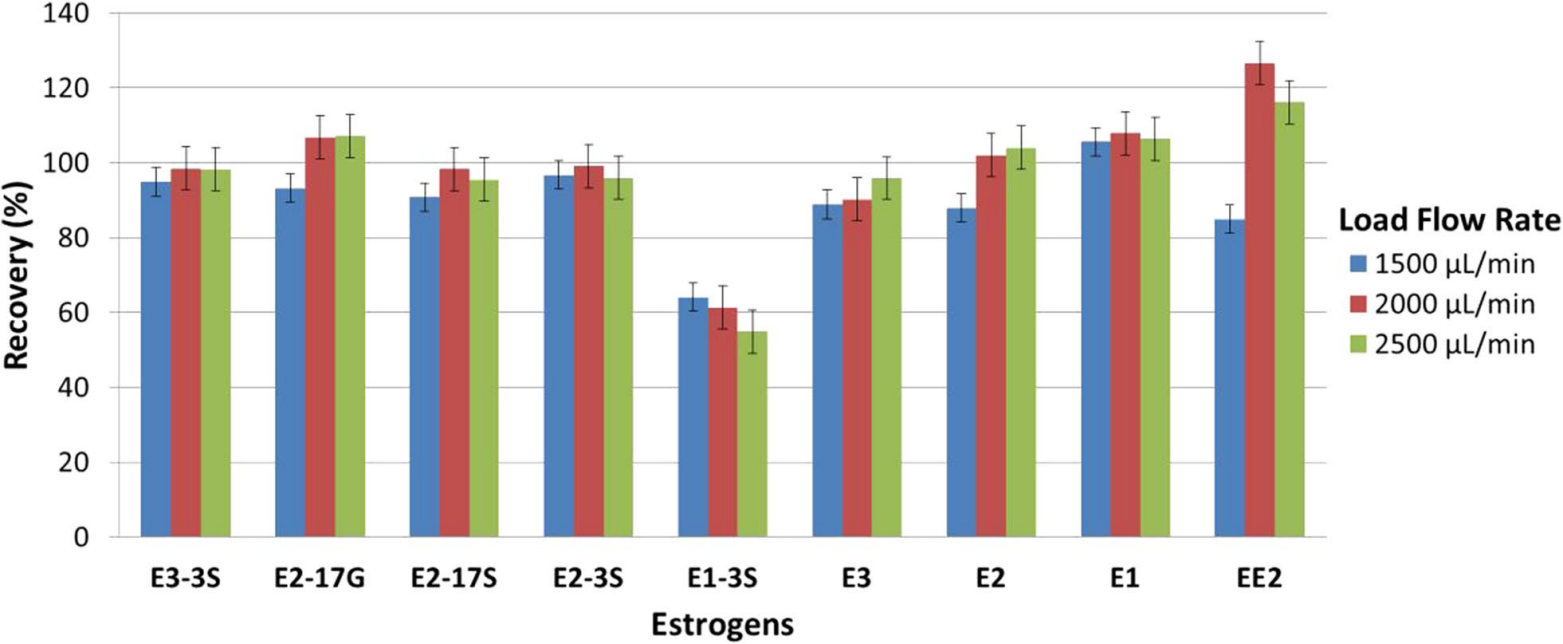
Effect of loading speed. Percentage recovery for all analytes tested using 1500 µl min^−1^, 2000 μL min^−1^ and 2500 µL min^−1^ flow rates. A flow of 1000 μl min^−1^ was considered as 100 % (n = 3, C = 500 ng L^−1^)

While performing solid-phase extraction, flow rates from 500 to 2500 μL min^−1^ were tested to evaluate the effect of loading speed. Load or elute flow rates that are too fast may not allow enough time for the analytes of interest to be bound or removed from the sorbent [30]. Absolute areas (without internal standard addition) for all target compounds were compared after analysis of a mix of compounds at 500 ng L^−1^ (data not shown). Although significant analyte loses were not observed even with a 2500 μl min^−1^ flow rate, (n = 3, C = 500 ng L^−1^, Fig. 3), very high flow rates could not be used given that excessive backpressure stopped the instrument. Therefore a loading flow rate of 1000 μL min^−1^ was chosen.

**Fig. 4:**
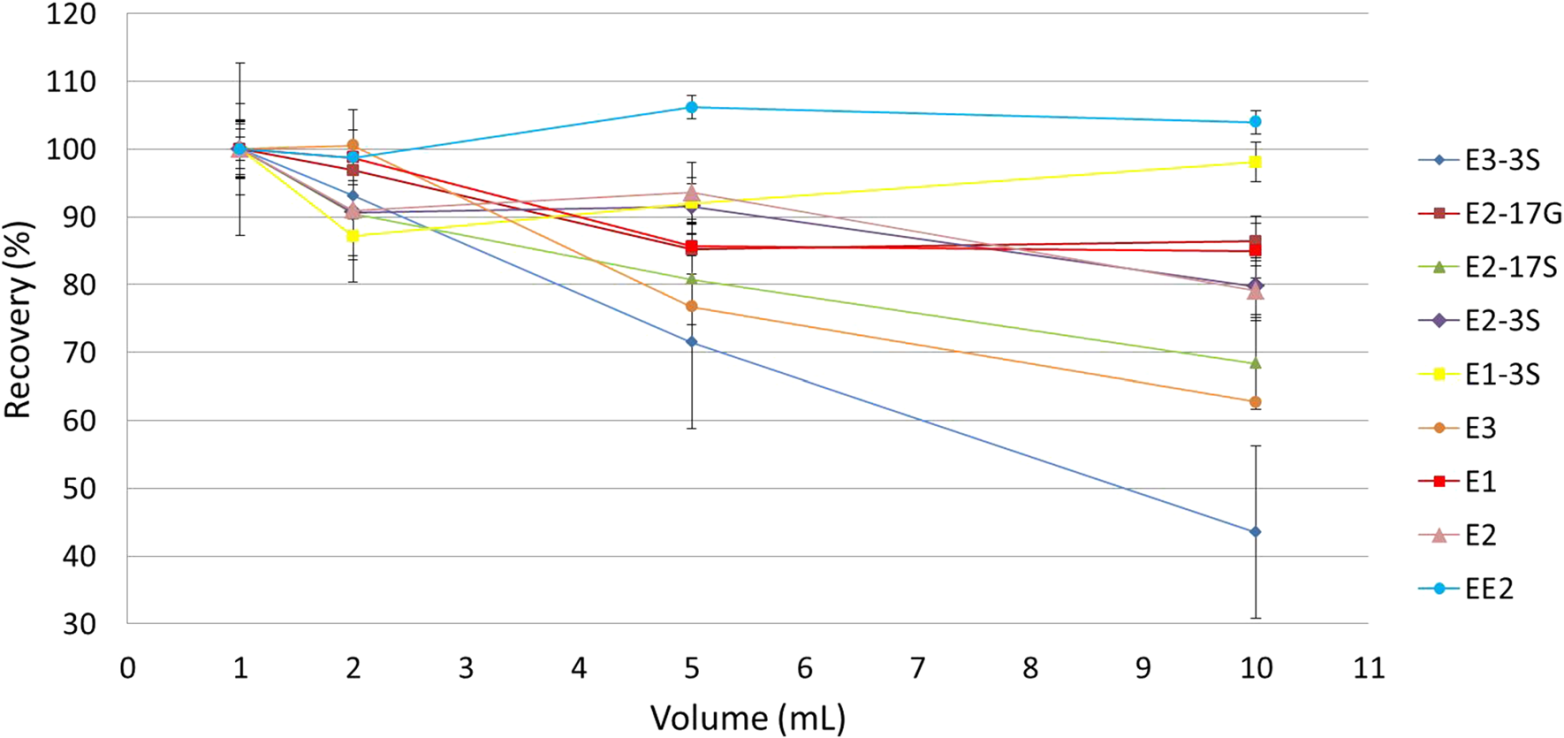
Breakthrough volume determination in HPLC water. Percentage recovery for 1, 2, 5 and 10 mL sample volume injections. 1 mL injection was considered as being 100 % (n = 3, C = 200 ng L^−1^)

The injection volume was evaluated to improve the method detection limits (MDLs) and signal intensities. A previous study showed that a pre-concentration of 10 mL sample could improve (MDLs) by a factor of 1.7–20 times compared to the same method using 1 mL injections [32]. Injections of 1, 2, 5 and 10 mL were tested (n = 3, C = 200 ng L^−1^) to evaluate the breakthrough volumes (Fig. 4). Results show that it is possible to use 5 mL sample injections without significant loss to almost all of the studied compounds while limiting the total analysis time. E3-3S and E3 compounds presented a little higher loss of signal at 5 mL (22 and 24 %, respectively), but since E3-3S is the compound that yields the best response to the method, the loss of the signal presented at 5 mL does not impair the results. In the case of E3, a compromise, accepting a higher analyte loss, was done once there was no significant loss to all other compounds analyzed. Higher injection volumes resulted in loss of analytes, possibly due to the presence of co-extracted substances during the loading step that may differentially affect the signal variability of each analyte. MDLs were obtained in the low ng L^−1^ range for all compounds which allowed the detection of trace amounts of the selected contaminants in all water matrices. Results obtained with 5 mL injections were lower by a factor of 0.8–10 times in HPLC water and 0.5–2.7 times in river water compared to 1 mL injections using exactly the same method. Sample size of 1 mL for wastewater samples were used due to the high matrix interference when 5 mL sample sizes were used.

Urine samples presented high concentrations for most of the studied conjugated estrogens. A dilution factor of ten was applied to urine sample before injecting a 1 mL aliquot. Thus, no other injection volume was tested for this matrix.

### Chromatographic analysis

Optimization of the chromatographic separation was done by a series of tests to study the behaviour of the system to variations of key parameters such as column type, solvent load flow rate, organic solvent type and column temperature.

Several mobile phase compositions were tested: acetonitrile (ACN) and water (H_2_O); ACN and H_2_O with 100 mM triethanolamine (TEA); ACN and H_2_O with 10 mM ammonium acetate; ACN and H_2_O with bicarbonate 10 mM [51]; methanol (MeOH) and H_2_O with 0.1 % NH_4_OH; MeOH and H_2_O with ethyl acetate 2, 5 and 10 %, 0.1 % NH_4_OH; MeOH and H_2_O. The optimal separation of the nine estrogens, presenting the best peak shape and separation was achieved using a binary mobile phase composed of 0.1 % NH_4_OH, H_2_O in combination with an organic mobile phase of 0.1 % NH_4_OH, MeOH.

**Fig. 5:**
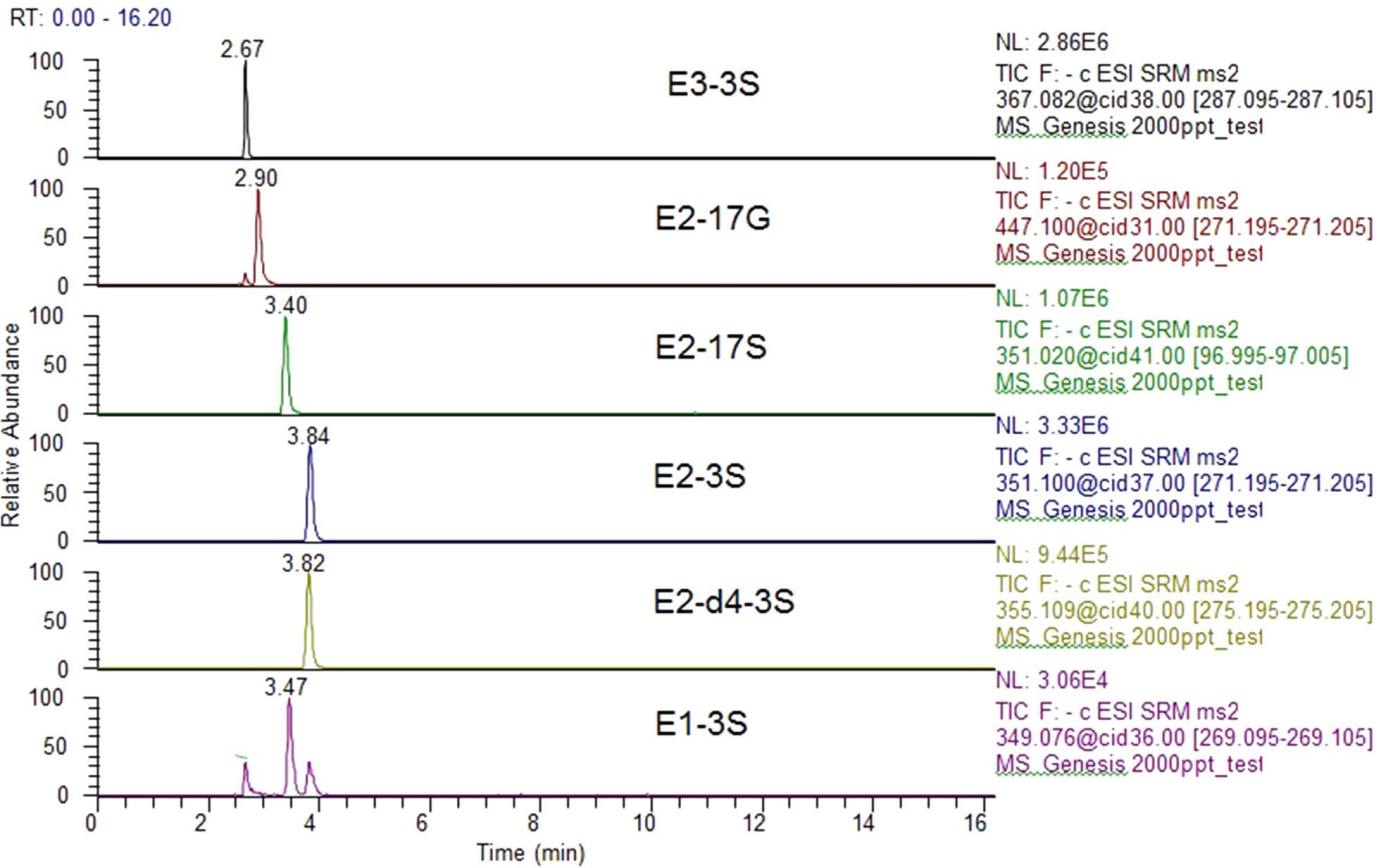
Representative chromatograms of a 2 μg L^−1^ standard mixture and of a 0.5 μg L^−1^ internal standard of the conjugated estrogens analyzed in river water

**Fig. 6:**
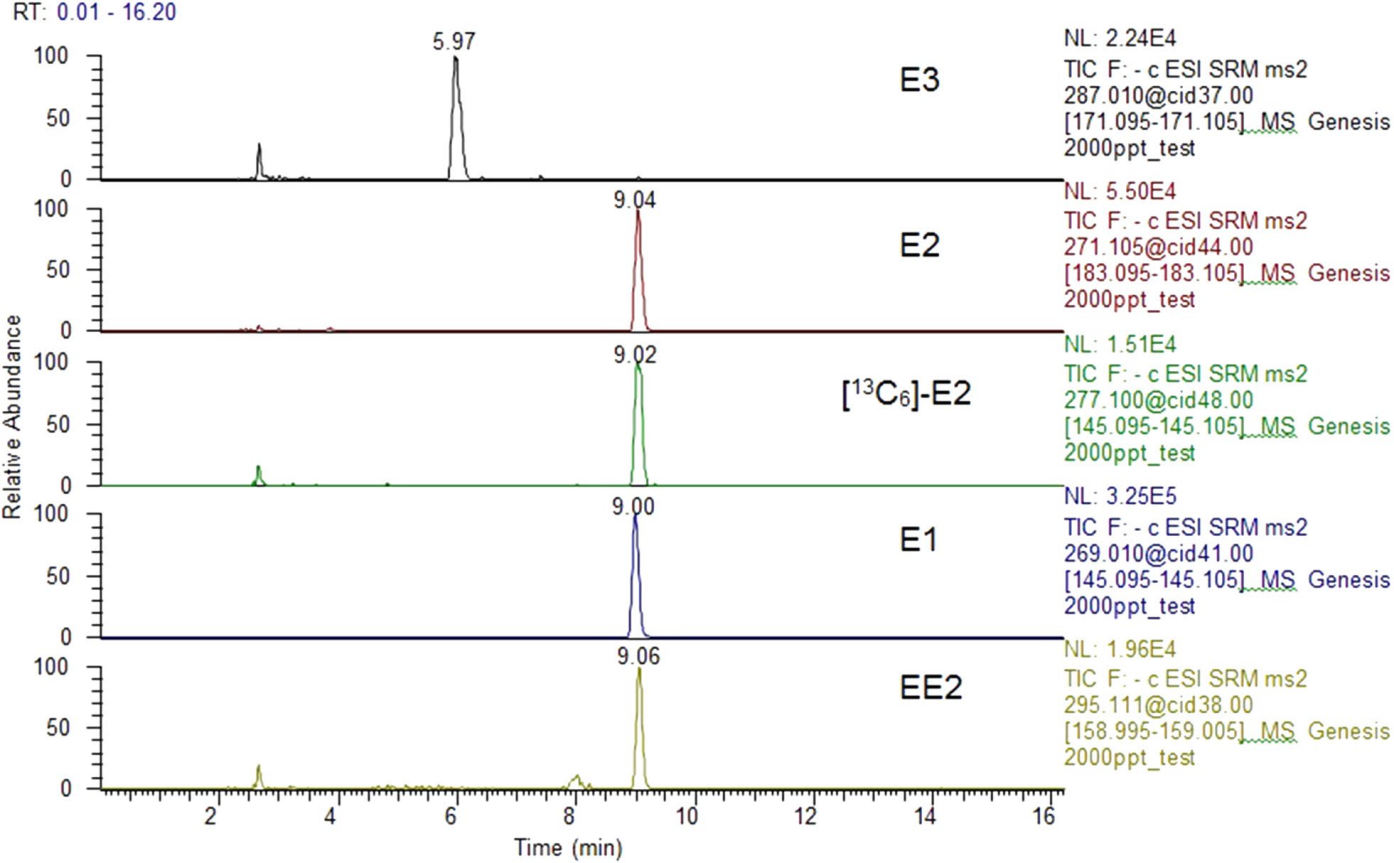
Representative chromatogram of a 2 μg L^−1^ standard mixture and of a 0.5 μg L^−1^ internal standard of the free estrogens analyzed in river water

Four different columns: Accucore RP-MS, 50 × 2.1 mm, 2.6 μm, Thermo Fisher Scientific, USA; Accucore RP-MS, 100 × 2.1 mm, 2.6 μm, Thermo Fisher Scientific, USA; Zorbax Extend-C18, Agilent, USA and BetaBasic Column C18, 100 × 2.1 mm, 3 µm, Thermo Fisher Scientific, USA were tested (results not shown). Similar results were found with 100 and 50 mm Accucore columns. BetaBasic Column C18 showed the best results. This column was chosen given its performance and to lower the possibility of peak broadening often observed when an on-line SPE column is coupled with an analytical column having a different type of solid phase chemistry [52]. Although many system configurations have been prone to premature aging of columns that do not survive more than a few dozens of analysis before columns need to be replaced given the pressure build up and column clogging [53], tests of the columns’ lifetime for our setup have shown that approximately 150 samples could be analyzed with the same column before significant changes were observed on peak shapes. Volume injections were set at 1 and 5 mL and the total time for analysis was 16 and 20 min respectively. Shorter times for separation were tested but resulted in co-elution for certain compounds. According to these results, the 10 min separation time for analysis was divided into two segments (conjugated and free estrogens) to improve sensitivity (Figs. 5, 6).

The optimal gradient elution program was a challenge given the similar structures of the estrogens and that some of them showed poor separation. Other studies presented the same limitations [34, 41]. Since tandem MS is used to detect the target compounds and they have different precursor ions and monitored transitions (Table 2), complete separation is not required. Final solvent flow rate was set to 250 μL min^−1^. Higher flow rates were tested but resulted in poor peak resolution and peak shapes (Fig. 3). Representative chromatograms of a 2 μg L^−1^ standard mixture of the compounds analyzed in river water are illustrated in Figs. 5 and 6.

Two internal standards (isotopically-labeled E2 and E2-3S) were used to compensate the signal reproducibility and variations between runs, for free and conjugated estrogens, respectively.

### Method validation

Validation data was obtained for all water matrices and a summary of the data is presented in Table 3. Additional files 2 and 3 also present the summary of the results obtained for precision.

**Table 3:** Limits of detection (LOD) in ng L^−1^ obtained for all water matrices tested

Estrogens	LOD (in ng L^−1^)^a^					
	HPLC 1 mL^b^	DW 1 mL^b^	RW 1 mL^b^	WW 1 mL^b^	HPLC 5 mL^b^	RW 5 mL^b^
E3-3S	7.1	13	7.1	41	9.2	6.3
E2-17G	27	21	48	42	14	21
E2-17S	6.9	17	8.2	28	4.7	3.3
E1-3S	25	63	74	76	4.6	27
E2-3S	8.9	14	5.0	13	3.4	5.3
E3	37	59	26	52	3.6	10
E2	19	14	9.7	14	6.1	9.5
E1	32	20	5.0	26	13	9.7
EE2	31	46	49	62	7.2	25

*DW* drinking water, *RW* river water, *WW* wastewater

^a^LOD—limit of detection, determined using the most abundant product ion

^b^Sample volume

Calibration curves were made using standard additions (Table 3 and Additional file 4) and show excellent determination coefficients (*R*^*2*^ > 0.993) for all the compounds in all tested matrices. Intra-day and inter-day precision were considered acceptable if lower than 20 % (Additional files 2, 3), while 30 % were acceptable for matrix interferences (accuracy) (Table 4) [48].

**Table 4:** Concentrations of the selected estrogens in the water samples analysed in ng L^−1^

Estrogens	Drinking water (UdeM)	Repentigny		St Lawrence river (Delson)	St Lawrence river (repentigny)	Prairie river	Thousand island river
		Wastewater	Effluent				
E3-3S	<7.1	<41	<6.3	<6.3	<6.3	<6.3	<6.3
E2-17G	<14	<42	<21	<21	<21	<21	<21
E2-17S	<4.7	<28	<3.3	<3.3	<3.3	<3.3	<3.3
E1-3S	<4.6	<76	<27	<27	<27	<27	<27
E2-3S	<3.4	<13	<5.3	<5.3	<5.3	<5.3	<5.3
E3	<3.6	<52	<10	<10	<10	<10	<10
E2	<6.1	<14	<9.5	<9.5	<9.5	<9.5	<9.5
E1	<13	<26	<9.7	<9.7	<9.7	<9.7	<9.7
EE2	<7.2	<62	<25	<25	<25	<25	<25

Samples were collected and analyzed in July 2014

In general, for water (HPLC, drinking water and river water), linearity was excellent with determination coefficients (R^2^ ≥ 0.991) for all target compounds. Method intra-day precision was between 3 and 14 % for 1 or 5 mL injection (C = 200 or 50 ng L^−1^; n = 10), except for E1-3S where results were 13–18 %. For inter-day precision results were lower than 20 % for 1 or 5 mL loops (C = 200 or 50 ng L^−1^; n = 12). A very low spike concentration (50 or 200 ng L^−1^) was used to perform validation tests and since E1-3S was the compound with the weakest signal in this method (Fig. 5), it was acceptable that it presented lower precision during the analysis. Consequently, even if all the results obtained are acceptable, validation data for this compound presented higher deviation results when compared with the data obtained for all the other target compounds. This limitation was not observed in samples with higher concentrations such as waste samples or urine.

Linearity for wastewater, was very good with determination coefficients (R^2^ ≥ 0.992), except for E3 for which R^2^ was 0.989 for 1 mL sample volume. Method intra-day precision was lower than 10 % (C = 200 ng L^−1^; n = 10) for all compounds except for E3 for which it was 18 % (n = 7) and lower than 20 % for inter-day precision (C = 200 ng L^−1^; n = 12).

For urine, linearity was excellent with determination coefficients varying between 0.991 ≤ R^2^ ≤ 0.999 for all the estrogens tested.

Extraction recovery results for all target compounds were good (>90 %). When lower spike concentration was used, extraction recoveries were generally good (>80 %), except for E3-3S and E1-3S (70.9 % for both compounds). Results are shown in Additional file 5. Extraction efficacies were tested in two different concentrations for 5 mL injections (C = 50 and 100 ng L^−1^; n = 7) and one concentration for 1 mL injections (C = 200 ng L^−1^; n = 10).

According to previous studies [34, 41], the possibility of sample carry over from repeat pre-concentration steps could cause significant concerns in on-line SPE methods. In order to prevent this, blanks (HPLC water without analytes or an internal standard solution) were extracted and analysed in duplicate in every sequence (begin, middle and end) as control for carry over and background concentrations. Blanks samples with internal standards were also analyzed during the analytical sequence to confirm the results. No carry over was noticed even when blanks were extracted and analyzed after 5000 ng L^−1^ spiked samples (results not shown).

Limits of detection (LOD) were evaluated in HPLC, drinking, river and wastewater. The most intense transition (SRM#1) was used to calculate the LOD, while the second most intense transition (SRM#2) was used to confirm the presence of the compound. The limit of detection (LOD) [48] ranged from 6.9 to 76 ng L^−1^ while the limit of quantification (LOQ) ranged from 21 to 228 ng L^−1^ for 1 mL volume injection. For 5 mL volume injection, the LOD ranged from 3.3 to 27 ng L^−1^ while the LOQ ranged from 10 to 81 ng L^−1^. Limits of detection and limits of quantification for all matrix tested are presented in Table 3. Additional files 6 and 7 present the results of this method compared to the detection limits and limits of quantification of others methods found in the literature. In general, the limits of detection of this method are around 10–100 times higher than the limits of detection found in the literature for wastewater samples analyzed by equivalent off-line methods. However, the amount of samples used to achieve these limits is 100–250 times lower. For river water, even if the amount of sample used is much lower (1–5 mL instead of 500–2000 mL in other methods), limits of detection are comparable in some cases. For E2, the detection limit for 5 mL samples is 9.5 ng L^−1^ while in some off-line method it is reported as 2.3 ng L^−1^ using 500 mL samples [47]. Similar results are observed for E1: 5 ng L^−1^, 1 mL sample, compared to 1.2 ng L^−1^ [47], 500 mL sample and E2-3S: 5.0 ng L^−1^, 5 mL sample, compared to 0.74 ng L^−1^[47] 500 mL sample, with LOD varying less than ten times to the online method described.

According to Garcia et al. [52] and Schuhmacher et al. [54] a major problem for quantitative analysis using HESI is the presence of matrix effects. Matrix effects are defined as the unexpected suppression or enhancement of the analyte response due to the presence of other compounds in the sample. Most of the compounds were not subjected to significant matrix effects (E2-17G, E2-17S, E2-3S, E1-3S, E2, E1 and EE2) while E3-3S was susceptible to signal enhancement and E3 to signal suppression. Results for matrix effects and accuracy are presented in Additional files 8 and 9. Some strategies to reduce matrix effects such as external calibration using matrix-matched samples, isotope dilution and standard additions have been recommended [55]. Although the addition of isotopically-labeled internal standards to compensate for matrix effects are often considered a lengthy and labor intensive method [28, 56]. The internal standards were used in this study since it was shown to be an efficient mean to correct signal distortion caused by matrix interferences.

The recovery of the urine samples using the online SPE method was evaluated at three different concentration levels (500, 1000 and 5000 ng L^−1^, n = 5). The mean peak areas of the selected estrogens in HPLC water for a 1 mL injection were compared with the same volume injection of those of urine samples for a dilution factor of ten. The same mass of analyte was injected in both cases. Results are shown in Additional file 10.

## Method application

### Analysis of drinking, river, wastewater and effluent water samples using on-line SPE–LC–ESI–MS

To demonstrate the applicability of the developed method, samples of drinking, river, sewage and effluent water from the region of Montreal, Quebec, Canada were analyzed. Results for water samples are summarized in Table 4.

Results show that free and conjugated estrogens were not found in concentrations above the LOD of the present method in drinking and river waters for Montreal area in Canada. In wastewater samples, estriol-3-sulfate (E3-3S) is most probably present in sewage and effluent samples, but with very low concentrations (lower than the method detection limit). Although a clear peak could be identified, the presence could not be confirmed by a second SRM transition. The absence of other targeted estrogens may be influenced by the choice of sampling sites. These levels were generally similar or lower to those previously reported [1, 2, 15, 23, 37, 44, 46, 47, 57]. In addition, most of the data for conjugated estrogens come from European rivers and wastewaters that present environmental conditions such as temperature and flow that are different from Montreal, QC, Canada.

Furthermore, in most methods found in the literature, large sample volumes (up to 4000 mL) are often used for solid phase extraction prior to analysis to detect estrogens [1, 15, 23, 37, 44–47, 57, 58]. However, the current method is efficient to quantitate and confirm estrogens (including conjugated forms) at low concentration levels (ng L^−1^) in complexes matrices such as river and wastewater sample using 1 and 5 mL injections. Table 5 for river water and Table 6 for wastewater show the concentrations found in the literature compared to the present on-line pre-concentration method.

**Table 5:** Comparison of reported concentrations of the studied estrogens in river samples

Estrogens	Present study	a	b	c	d
E3-3S	<6.3	<0.3	NA	ND	<0.07
E2-17G	<21	<3.1	<2.24	ND	1.10–7.34
E2-17S	<3.3	NA	NA	NA	NA
E1-3S	<25	0.3–0.8	ND–7	0.3–7	<0.16
E2-3S	<5.3	0.2–0.8	NA	0.2–0.4	0.59–0.85
E3	<10	NA	NA	ND–51	1–7.27
E2	<9.5	NA	NA	ND–8.8	ND
E1	<9.7	0.2–6.6	4–22	<0.1–17	ND
EE2	<25	NA	NA	NA	ND

Concentrations in ng L^−1^

*NA* not analyzed

*ND* not detected

*a* Isobe et al. [44], 1000 mL volume sample

*b* Mozaz et al. [46], 500 mL volume sample

*c* Liu et al. [1], no information about volume sample

*d* Kuster et al. [47], 500 mL volume sample

**Table 6:** Comparison of measured concentrations of the studied estrogens in wastewater samples (in ng L^−1^)

Estrogens	Present study		a		c		e		f		g		h	
	WW	Eff	WW	Eff	WW	Eff	WW	Eff	WW	Eff	WW	Eff	WW	Eff
E3-3S	<41	<6.3	NA	<0.3	6.5–333	0.6–160	<1.6	<0.42	NA	NA	14	14	NA	NA
E2-17G	<51	<21	NA	<3.1	ND	ND	<1.7	<0.52	NA	NA	<3	<3	NA	NA
E2-17S	<28	<3.3	NA	NA	NA	NA	NA	NA	NA	NA	NA	NA	NA	NA
E1-3S	<76	<27	NA	0.3–2.2	1.2–170	ND–42	2.9	3.9	10	12	25	25	NA	NA
E2-3S	<13	<5.3	NA	<0.2–1.0	3.2–957	ND–94	<1.1	<0.22	NA	NA	3.3	3.3	NA	NA
E3	<52	<10	NA	NA	ND–660	ND–275	100	ND	50	1.0	33–187	0.43–18	74–234	46–175
E2	<14	<9.5	NA	NA	ND–162	ND–158	2	ND	5.0	0.7	4–25	0.55–3.3	ND–74	ND–51
E1	<26	<9.7	NA	2.5–34	ND–670	ND–147	100	5	15	3.0	25–132	2.5–82	ND–376	ND–42
EE2	<62	<25	NA	NA	NA	NA	15	5	1.2	1.0	0.43–13	ND–1	ND	ND

Concentrations in ng L^−1^

*NA* not analyzed

*ND* not detected

*Eff* effluent

*WW* wastewater

*a* Isobe et al. [44], 1000 mL volume sample

*c* Liu et al. [1], no information about volume sample

*e* Gentili et al. [37], 2000 mL river, 250 mL effluent and 100 wastewater volume sample

*f* Koh et al. [38], 1000 mL volume sample

*g* Baronti et al. [15], 400 mL wastewater and 150 mL wastewater volume sample

*h* Fayad [39], 10 mL volume sample

### Determination of conjugated and free estrogen levels in female urine samples using on-line SPE–LC–HESI–MS

Zhang and Henion [59] and D’Asenzo [57] showed that LC–MS/MS, can be successfully used for determining the low levels of estrogen sulfates in female urine. By using a similar technique, but with an online SPE extraction, an increased number of conjugated estrogens excreted in female urine have been observed. All the conjugated estrogens analyzed were identified. Regarding the free estrogens, apart from some E3 in the urine of pregnant women, they were never detected.

The complete data on amounts of estrogens in urine of women (pregnant or not) are presented in Table 7. The results are similar to those previously measured in other studies [1], however it is difficult to compare given that many such studies are based on daily excretion and not on urine concentration (the results are usually in micrograms per day and not in micrograms per liter). As expected, estrogen levels in the urine of pregnant women were much higher than in the urine of non-pregnant women of similar age.

**Table 7:** Concentrations of the selected estrogens in the urine samples analysed in µg L^−1^

Estrogens	LOD (drinking water)	Pregnant women			Women		
		A (40 years old)	B (30 years old)	C (25 years old)	D (30 years old)	E (35 years old)	F (15 year old)
E3-3S	0.001	493	577	988	16.9	22.5	10.8
E2-17G	0.001	662	798	1707	4.834	10.9	2.29
E2-17S	0.005	<0.005	<0.005	<0.005	6.71	6.68	7.91
E1-3S	0.005	5332	9750	2950	36.2	30.9	NA
E2-3S	0.003	10.1	16.5	5.36	1.74	0.473	2.97
E3	0.004	2.09	1.22	14.2	<0.004	<0.004	<0.004
E2	0.006	<0.006	<0.006	<0.006	<0.006	<0.006	<0.006
E1	0.013	0.42	<0.013	1.08	<0.013	<0.013	<0.013
EE2	0.007	<0.007	<0.007	<0.07	<0.007	<0.007	<0.007

Samples were collected and analyzed in September and October 2014

## Conclusion

An on-line SPE LC/MS/MS method for the simultaneous determination and quantification of conjugated and free hormones was developed and validated for the analysis of urine samples, drinking and surface water samples, as well as sewage and wastewater effluent samples. Contrary to published methods using large sample volumes (about 250 mL–4 L) and time-consuming offline SPE, we were able to quantitate all the proposed hormones using a small sample volume (1–5 mL). All the compounds could be determined at low nanogram-per-liter range (3–15 ng L^−1^) with a recovery higher than 70 % for all the compounds in all water matrices. For urine samples, limits of detection ranged from 30 to 150 ng L^−1^ since the expected concentrations were much higher and they were diluted at least ten times to avoid matrix interferences. Samples were analyzed in <20 min runs, with only 10 min for analytes separation without the time-consuming steps required for the standard off-line SPE methods. The main advantage of the on-line SPE is that manual sample preparation was limited to sample filtration and spiking of the internal standard solution. This eliminates several working steps, such as extraction, evaporation and reconstitution, and significantly reduces time and procedural errors.

Method detection limits of the nine hormones ranged from 3 to 15 ng L^−1^ in clean water but were limited to 14 to 76 ng L^−1^ in wastewater samples. For all analytes, method intra-day and inter-day precision were less than 20 %. Accuracy was ±30 %. Such MDL are excellent for urine analysis but will only be useful in environmental analysis for fairly contaminated samples or for experimental designs where compounds are spiked.

The results show that the presented method can potentially be applied to the simultaneous analysis of the conjugated and free estrogens at low nanogram-per-liter levels in complex water matrices and urine samples even if further optimization of the method for preconcentration could be necessary to improve quantification limits for clean environmental samples. Considering that the presented method is able to quantitate both conjugated and free species of estrogens, in the same run without any particular preparation, it also shows potential for studying the deconjugation of metabolized estrogens in the contaminated water matrices and their implication on the environmental fate of estrogens, especially considering the fate of conjugated hormones from urine.

## Additional files

10.1186/s13065-016-0174-z Valve program, on-line SPE (loading pump) and LC (analytical pump). Gradient elution program for 1 and 5 mL injections, used for the pre-concentration and separation of selected estrogens. Solvents consist of: H_2_O with 0.1 % NH_4_OH(A) and MeOH with 0.1 % NH_4_OH (B).
10.1186/s13065-016-0174-z Method validation for precision (intra-day). C = 200 ng L^−1^, n = 10 for 1 mL sample volume and C = 50 ng L^−1^, n = 7 for 5 mL sample volume.
10.1186/s13065-016-0174-z Method validation for precision (inter-day). C = 200 ng L^−1^, n = 12 for 1 mL sample volume and C = 50 ng L^−1^, n = 15 for 5 mL sample volume.
10.1186/s13065-016-0174-z Method validation results for linearity (R^2^), for all waters tested (HPLC water, drinking water, river water and wastewater).
10.1186/s13065-016-0174-z Extraction recovery results for all target compounds in river water. Extraction efficacies were tested in two different concentrations for 5 mL injections (C = 50 and 100 ng L^−1^; n = 7) and one concentration for 1 mL injections (C = 200 ng L^−1^; n = 10).
10.1186/s13065-016-0174-z Comparison of measured detection limits (LODs) of the studied estrogens with other methods found in the literature for water samples. Concentrations in ng L^−1^.
10.1186/s13065-016-0174-z Comparison of measured quantification limits (LOQs) of the studied estrogens with other methods found in the literature for water samples. Concentrations in ng L^−1^.
10.1186/s13065-016-0174-z Accuracy for the selected estrogens for all waters tested.
10.1186/s13065-016-0174-z Matrix Effects for the selected estrogens for all waters tested (in percentage).
10.1186/s13065-016-0174-z Calculated recovery values in percentage for the selected estrogens. BetaBasic column was used as SPE column for the on-line SPE–LC–MS/MS method. Recovery values were calculated comparing the same volume injection of those of urine samples diluted at least ten times. (n = 5).

## Abbreviations

ACN: acetonitrile
APCI: atmospheric-pressure chemical ionization
C_blank_: concentration in the blank sample
C_e_: expected concentration
C_m_: measured concentration
C_standard_: concentration in the spiked sample
[^13^-C_6_]-E2: [^13^-C_6_]-estradiol
DW: drinking water
E1: estrone
E1-3S: estrone-3-sulfate
E2: estradiol
E2-17G: estradiol-17-glucoronide
E2-17S: estradiol-17-sulfate
E2-3S: estradiol-3-sulfate
E2-d4-3S: estradiol-d4-3-sulfate
E3: estriol
E3-3S: estriol-3-sulfate
EDCs: endocrine disrupting compounds
EE2: 17-alpha-ethinylestradiol
EEC: European Commission, Council Regulation
Eff: efflent
GC–MS: gas chromatography–mass spectrometry
H_2_O: water
HESI: heated electrospray ionization
HPLC: high performance liquid chromatography
LC: liquid chromatography
LOQ: quantification limit
MDL: method detection limit
MeOH: methanol
MS/MS: tandem mass spectrometry
NA: not analyzed
ND: not detected
NH_4_OH: ammonium hydroxide
NI: negative ionization mode
QC: quality control
(QC): Quebec
R^2^: determination coefficient
RSD: relative standard deviation
RW: river water
SD: standard deviation
SPE: solid phase extraction
SRM: selected reaction-monitoring mode
TEA: triethanolamine
WW: wastewater
WWTP: wastewater treatment plant
