# Supplementary material for: Analysis of steroid hormones and their conjugated forms in water and urine by on-line solid-phase extraction coupled to liquid chromatography tandem mass spectrometry
Source: Chem Cent J. 2016 May 6;10:30. doi: 10.1186/s13065-016-0174-z (PMC4859969; doi:10.1186/s13065-016-0174-z)
Supplement: Supplementary file 1 — 10.1186/s13065-016-0174-z Valve program, on-line SPE (loading pump) and LC (analytical pump). Gradient elution program for 1 and 5 mL injections, used for the pre-concentration and separation of selected estrogens. Solvents consist of: H2O with 0.1 % NH4OH(A) and MeOH with 0.1 % NH4OH (B). [file 13065_2016_174_MOESM1_ESM.docx]

Table 1 – Valve program, on-line SPE (loading pump) and LC (analytical pump).

Gradient elution program for 1 mL and 5 mL injections, used for the pre-concentration and separation of selected estrogens. Solvents consist of: H_2_O with 0.1% NH_4_OH(A) and MeOH with 0.1% NH_4_OH (B).
