# Supplementary material for: Analysis of steroid hormones and their conjugated forms in water and urine by on-line solid-phase extraction coupled to liquid chromatography tandem mass spectrometry
Source: Chem Cent J. 2016 May 6;10:30. doi: 10.1186/s13065-016-0174-z (PMC4859969; doi:10.1186/s13065-016-0174-z)
Supplement: Supplementary file 2 — 10.1186/s13065-016-0174-z Method validation for precision (intra-day). C = 200 ng L−1, n = 10 for 1 mL sample volume and C = 50 ng L−1, n = 7 for 5 mL sample volume. [file 13065_2016_174_MOESM2_ESM.docx]

Figure 1 – Method validation for precision (intra-day). C = 200 ng L^-1^, n = 10 for 1 mL sample volume and C = 50 ng L^-1^, n = 7 for 5 mL sample volume.
