# Supplementary material for: Analysis of steroid hormones and their conjugated forms in water and urine by on-line solid-phase extraction coupled to liquid chromatography tandem mass spectrometry
Source: Chem Cent J. 2016 May 6;10:30. doi: 10.1186/s13065-016-0174-z (PMC4859969; doi:10.1186/s13065-016-0174-z)
Supplement: Supplementary file 3 — 10.1186/s13065-016-0174-z Method validation for precision (inter-day). C = 200 ng L−1, n = 12 for 1 mL sample volume and C = 50 ng L−1, n = 15 for 5 mL sample volume. [file 13065_2016_174_MOESM3_ESM.docx]

Figure 2 - Method validation for precision (inter-day). C = 200 ng L^-1^, n = 12 for 1 mL sample volume and C = 50 ng L^-1^, n = 15 for 5 mL sample volume.

Table ST-2 – Extraction recovery results for all target compounds in river water. Extraction efficacies were tested in two different concentrations for 5 mL injections (C = 50 ng L^-1^ and 100 ng L^-1^; n=7) and one concentration for 1 mL injections (C = 200 ng L^-1^; n=10).

|  | **Extraction Recoveries (%)** | | |
| --- | --- | --- | --- |
|  | 1 mL injection | 5 mL injection | 5 mL injection |
|  | C= 200 ngL^-1^ | C= 50 ngL^-1^ | C= 100 ngL^-1^ |
| **E3-3S** | 97.7 | 70.9 | 104 |
| **E2-17G** | 94.1 | 84.0 | 98.4 |
| **E2-17S** | 98.4 | 92.3 | 121 |
| **E1-3S** | 102 | 70.9 | 104 |
| **E2-3S** | 98.0 | 93.8 | 98.4 |
| **E3-3S** | 101 | 91.7 | 122 |
| **E2** | 102 | 85.4 | 103 |
| **E1-3S** | 101 | 94.6 | 101 |
| **EE2** | 111 | 93.6 | 97.3 |

Table ST-3 – Comparison of measured detection limits (LODs) of the studied estrogens with other methods found in the literature for water samples. Concentrations in ng L^-1^.

| **Estrogens** | **MDL ^(a)^** |  |  |  |  |  |  |  |  |  |  |  |  |  |
| --- | --- | --- | --- | --- | --- | --- | --- | --- | --- | --- | --- | --- | --- | --- |
|  | Present Method | | | | | | A | | B | | | C | D | E |
|  | HPLC | DW ^(c)^ | RW ^(d)^ | WW ^(e)^ | HPLC | RW ^(d)^ | HPLC | RW ^(d)^ | WW^(e)^ | Eff^(f)^ | RW | GW^(g)^ | N/D * | N/D |
|  | 1 mL^(b)^ | 1 mL^(b)^ | 1 mL^(b)^ | 1 mL^(b)^ | 5 mL^(b)^ | 5 mL^(b)^ | 500 mL ^(b)^ | 500 mL ^(b)^ | 100 mL^(b)^ | 250 mL ^(b)^ | 2000 mL ^(b)^ | 500 mL ^(b)^ | N/D * | 1000 mL^(b)^ |
| **E3-3S** | 7.1 | 13 | 7.1 | 41 | 9.2 | 6.3 | 0.04 | 0.07 | 1.6 | 0.42 | 0.05 | N/A | N/A | 0.3 |
| **E2-17G** | 27 | 21 | 48 | 42 | 14 | 21 | 0.23 | 0.74 | 1.7 | 0.52 | 0.06 | 2.24 | 0.005 | 3.1 |
| **E2-3S** | 8.9 | 14 | 5.0 | 13 | 3.4 | 5.3 | 0.23 | 0.74 | 1.1 | 0.22 | 0.03 | N/A | N/A | 0.2 |
| **E1-3S** | 25 | 63 | 74 | 76 | 4.6 | 27 | 0.36 | 0.16 | 0.2 | 0.04 | 0.005 | 0.53 | 0.0001 | 0.1 |
| **E2-17S** | 6.9 | 17 | 8.2 | 28 | 4.7 | 3.3 | N/A | N/A | N/A | N/A | N/A | N/A | N/A | N/A |
| **E1** | 32 | 20 | 5.0 | 26 | 13 | 9.7 | 0.57 | 1.15 | 0.4 | 0.04 | 0.005 | 2.5 | 1 | 0.1 |
| **E2** | 19 | 14 | 9.7 | 14 | 6.1 | 9.5 | 1.22 | 2.27 | 0.65 | 0.16 | 0.02 | 2.5 | 1 | 0.3 |
| **EE2** | 31 | 46 | 49 | 62 | 7.2 | 25 | 1.51 | 7.55 | N/A | N/A | N/A | 3.22 | 2 | 0.2 |
| **E3** | 37 | 59 | 26 | 52 | 3.6 | 10 | 0.41 | 1.13 | 0.85 | 0..24 | 0.03 | 5.04 | 1 | 1.5 |

(a)LOD - Limit of detection, determined using the most abundant product ion.

(b) Sample volume.

(c) DW - Drinking water; (d) RW - River water; (e) WW - Wastewater; (f) Eff - Effluent; (g) GW - Groundwater.

N/A - Not analyzed

N/D - No Information

* IDL - Instrument detection limit

A – Kuster et al.[[43](#_ENREF_43)];

B – Gentili et al.[[33](#_ENREF_33)];

C – Mozaz et al.[[42](#_ENREF_42)];

D – Díaz-Cruz et al. [[2](#_ENREF_2)];

E – Isobe et al.[[40](#_ENREF_40)];

Table ST-4 – Comparison of measured quantification limits (LOQs) of the studied estrogens with other methods found in the literature for water samples. Concentrations in ng L^-1^.

| **Estrogens** | **LOQ ^(a)^** |  |  |  |  |  |  |  |  |  |  |  |
| --- | --- | --- | --- | --- | --- | --- | --- | --- | --- | --- | --- | --- |
|  | Present Method | | | | | | F | | | G | | |
|  | HPLC | DW ^(c)^ | RW ^(d)^ | WW ^(e)^ | HPLC | RW ^(d)^ | WW ^(e)^ | WW^(e)^ | Eff^(f)^ | WW^(e)^ | Eff^(f)^ | RW ^(d)^ |
|  | 1 mL^(b)^ | 1 mL^(b)^ | 1 mL^(b)^ | 1 mL^(b)^ | 5 mL^(b)^ | 5 mL^(b)^ | 50 mL ^(b)^ | 100 mL^(b)^ | 250 mL ^(b)^ | 150 mL^(b)^ | 400 mL^(b)^ | 4000 mL^(b)^ |
| **E3-3S** | 21 | 39 | 21 | 123 | 28 | 19 | 5 | 3 | 1 | N/A | N/A | N/A |
| **E2-17G** | 81 | 63 | 144 | 126 | 42 | 63 | 6 | 3 | 1 | N/A | N/A | N/A |
| **E2-3S** | 27 | 42 | 15 | 39 | 10 | 16 | 3 | 2 | 1 | N/A | N/A | N/A |
| **E1-3S** | 75 | 189 | 222 | 228 | 14 | 81 | 2 | 0.8 | 0.3 | N/A | N/A | N/A |
| **E2-17S** | 21 | 51 | 25 | 84 | 14 | 9.9 | N/A | N/A | N/A | N/A | N/A | N/A |
| **E1** | 96 | 60 | 15 | 78 | 39 | 29 | 2 | 1 | 0.5 | 0.2 | 0.08 | 0.008 |
| **E2** | 57 | 42 | 29 | 42 | 18 | 29 | 4 | 2 | 1 | 0.6 | 0.2 | 0.02 |
| **EE2** | 93 | 138 | 147 | 186 | 22 | 75 | N/A | N/A | N/A | 0.9 | 0.3 | 0.03 |
| **E3** | 111 | 177 | 78 | 156 | 11 | 30 | 4 | 2 | 1 | 0.6 | 0.2 | 0.02 |

(a)LOQ - Limit of Quantification, determined using the most abundant product ion.

(b) Sample volume.

(c) DW - Drinking water; (d) RW - River water; (e) WW - Wastewater; (f) Eff - Effluent.

N/A - Not analyzed

F - D’Asenzo et al. [[52](#_ENREF_52)];

G - Baronti et al.[[15](#_ENREF_15)];

Table ST-5 – Accuracy for the selected estrogens for all waters tested.

**Accuracy ^(a)^**

| Estrogens | DW ^(b)^ | RW ^(c)^ | WW ^(d)^ | WW* ^(d)^ | RW** ^(c)^ |
| --- | --- | --- | --- | --- | --- |
|  | 1 mL^(e)^ | 1 mL^(e)^ | 1 mL^(e)^ | 1 mL^(e)^ | 5 mL^(e)^ |
| E3-3S | 18 | -2.3 | 70 | 30 | -4.5 |
| E2-17G | 7.9 | -5.9 | -3.9 | -11 | 4.1 |
| E2-17S | 7.1 | -1.6 | 8.0 | 0.4 | -6.2 |
| E1-3S | 1.1 | 2.4 | X | -0.1 | -4.5 |
| E2-3S | 4.5 | -2.0 | 3.9 | -2.5 | -9.4 |
| E3 | 7.3 | 0.8 | -75 | -36 | -6.2 |
| E2 | 4.5 | 2.7 | 0.7 | 4.3 | -11 |
| E1 | 0.5 | 0.6 | -15 | -5.5 | -6.9 |
| EE2 | 7.2 | 11 | 2 | 0.2 | 1.2 |

(a) Accuracy for the selected estrogens (C = 200 ng L^-1^, n = 10). * (C = 1000 ng L^-1^, n = 10). ** (C = 50 ng L^-1^, n = 10).

(b) DW - drinking water; (c) RW - river water; (d) WW – wastewater (e) Sample volume.

Table ST-6 – Matrix Effects for the selected estrogens for all waters tested (in percentage).

| **Estrogens^(a)^** | **DW ^(b)^** | **RW ^(c)^** | **RW* ^(c)^** | **WW ^(d)^** |
| --- | --- | --- | --- | --- |
|  | **1 mL^(e)^** | **1 mL^(e)^** | **5 mL^(e)^** | **1 mL^(e)^** |
| E3-3S | 111 | 85 | 84 | 153 |
| E2-17G | 90 | 91 | 107 | 87 |
| E2-17S | 100 | 93 | 97 | 98 |
| E1-3S | 99 | 100 | 154 | 148 |
| E2-3S | 128 | 120 | 95 | 78 |
| E3 | 113 | 107 | 91 | 120 |
| E2 | 117 | 113 | 99 | 103 |
| E1 | 95 | 99 | 100 | 78 |
| EE2 | 137 | 141 | 86 | 94 |

(a) Matrix effects for the selected estrogens (C = 200 ng L^-1^, n = 10). * (C = 50 ng L^-1^, n = 10).

(b) DW - drinking water; (c) RW - river water; (d) WW – wastewater (e) Sample volume.

Table ST-7 – Calculated recovery values in percentage for the selected estrogens. BetaBasic column was used as SPE column for the on-line SPE-LC-MS/MS method. Recovery values were calculated comparing the same volume injection of those of urine samples diluted at least ten times. (n = 5).

| Estrogens | Recovery (%) | | |
| --- | --- | --- | --- |
|  | 500 ng L^-1^ | 1000 ng L^-1^ | 5000 ng L^-1^ |
| E3-3S | 85 | 83 | NC |
| E2-17G | 94 | 90 | NC |
| E2-17S | 81 | 96 | 63 |
| E1-3S | 87 | 73 | NC |
| E2-3S | 92 | 93 | 64 |
| E3 | 114 | 109 | 115 |
| E2 | 117 | 99 | 97 |
| E1 | 110 | 94 | 94 |
| EE2 | 118 | 99 | 97 |

NC – not calculated because the signal was too strong
