# Supplementary material for: Analysis of steroid hormones and their conjugated forms in water and urine by on-line solid-phase extraction coupled to liquid chromatography tandem mass spectrometry
Source: Chem Cent J. 2016 May 6;10:30. doi: 10.1186/s13065-016-0174-z (PMC4859969; doi:10.1186/s13065-016-0174-z)
Supplement: Supplementary file 4 — 10.1186/s13065-016-0174-z Method validation results for linearity (R2), for all waters tested (HPLC water, drinking water, river water and wastewater). [file 13065_2016_174_MOESM4_ESM.docx]

Table 2 – Method validation results for linearity (R^2^), for all waters tested (HPLC water, drinking water, river water and wastewater).

| **Estrogens** | **R^2 (a)^** |  |  |  |  |  |
| --- | --- | --- | --- | --- | --- | --- |
|  | HPLC  1 mL^(b)^ | DW ^(c)^  1 mL^(b)^ | RW ^(d)^  1 mL^(b)^ | WW ^(e)^  1 mL^(b)^ | HPLC  5 mL^(b)^ | RW ^(d)^  5 mL^(b)^ |
| E3-3S | 0.9979 | 0.996 | 0.9984 | 0.9915 | 0.9946 | 0.9949 |
| E2-17G | 0.9921 | 0.9966 | 0.9947 | 0.9963 | 0.9953 | 0.9960 |
| E2-17S | 0.9982 | 0.9975 | 0.9989 | 0.9921 | 0.9959 | 0.9991 |
| E1-3S | 0.9925 | 0.9929 | 0.9959 | 0.9946 | 0.9963 | 0.9885 |
| E2-3S | 0.9976 | 0.9947 | 0.9989 | 0.9937 | 0.9953 | 0.9917 |
| E3 | 0.9932 | 0.9908 | 0.9978 | 0.9888 | 0.9958 | 0.9983 |
| E2 | 0.9963 | 0.9914 | 0.9990 | 0.9954 | 0.9951 | 0.9939 |
| E1 | 0.9972 | 0.9964 | 0.9987 | 0.9959 | 0.9947 | 0.9927 |
| EE2 | 0.9953 | 0.9928 | 0.9971 | 0.9935 | 0.9948 | 0.9933 |

(a) R^2^ determined by internal standard calibration for spiked solution (n=3, at least 6 point calibration curve).

(b) Sample volume.

(c) DW - Drinking water; (d) RW - River water; (e) WW - Wastewater
