# Supplementary material for: Analysis of steroid hormones and their conjugated forms in water and urine by on-line solid-phase extraction coupled to liquid chromatography tandem mass spectrometry
Source: Chem Cent J. 2016 May 6;10:30. doi: 10.1186/s13065-016-0174-z (PMC4859969; doi:10.1186/s13065-016-0174-z)
Supplement: Supplementary file 5 — 10.1186/s13065-016-0174-z Extraction recovery results for all target compounds in river water. Extraction efficacies were tested in two different concentrations for 5 mL injections (C = 50 and 100 ng L−1; n = 7) and one concentration for 1 mL injections (C = 200 ng L−1; n = 10). [file 13065_2016_174_MOESM5_ESM.docx]

Table 3 – Extraction recovery results for all target compounds in river water. Extraction efficacies were tested in two different concentrations for 5 mL injections (C = 50 ng L^-1^ and 100 ng L^-1^; n=7) and one concentration for 1 mL injections (C = 200 ng L^-1^; n=10).

|  | **Extraction Recoveries (%)** | | |
| --- | --- | --- | --- |
|  | 1 mL injection | 5 mL injection | 5 mL injection |
|  | C= 200 ngL^-1^ | C= 50 ngL^-1^ | C= 100 ngL^-1^ |
| **E3-3S** | 97.7 | 70.9 | 104 |
| **E2-17G** | 94.1 | 84.0 | 98.4 |
| **E2-17S** | 98.4 | 92.3 | 121 |
| **E1-3S** | 102 | 70.9 | 104 |
| **E2-3S** | 98.0 | 93.8 | 98.4 |
| **E3-3S** | 101 | 91.7 | 122 |
| **E2** | 102 | 85.4 | 103 |
| **E1-3S** | 101 | 94.6 | 101 |
| **EE2** | 111 | 93.6 | 97.3 |
