# Supplementary material for: Analysis of steroid hormones and their conjugated forms in water and urine by on-line solid-phase extraction coupled to liquid chromatography tandem mass spectrometry
Source: Chem Cent J. 2016 May 6;10:30. doi: 10.1186/s13065-016-0174-z (PMC4859969; doi:10.1186/s13065-016-0174-z)
Supplement: Supplementary file 6 — 10.1186/s13065-016-0174-z Comparison of measured detection limits (LODs) of the studied estrogens with other methods found in the literature for water samples. Concentrations in ng L−1. [file 13065_2016_174_MOESM6_ESM.docx]

Table 4 – Comparison of measured detection limits (LODs) of the studied estrogens with other methods found in the literature for water samples. Concentrations in ng L^-1^.

| **Estrogens** | **MDL ^(a)^** |  |  |  |  |  |  |  |  |  |  |  |  |  |
| --- | --- | --- | --- | --- | --- | --- | --- | --- | --- | --- | --- | --- | --- | --- |
|  | Present Method | | | | | | A | | B | | | C | D | E |
|  | HPLC | DW ^(c)^ | RW ^(d)^ | WW ^(e)^ | HPLC | RW ^(d)^ | HPLC | RW ^(d)^ | WW^(e)^ | Eff^(f)^ | RW | GW^(g)^ | N/D * | N/D |
|  | 1 mL^(b)^ | 1 mL^(b)^ | 1 mL^(b)^ | 1 mL^(b)^ | 5 mL^(b)^ | 5 mL^(b)^ | 500 mL ^(b)^ | 500 mL ^(b)^ | 100 mL^(b)^ | 250 mL ^(b)^ | 2000 mL ^(b)^ | 500 mL ^(b)^ | N/D * | 1000 mL^(b)^ |
| **E3-3S** | 7.1 | 13 | 7.1 | 41 | 9.2 | 6.3 | 0.04 | 0.07 | 1.6 | 0.42 | 0.05 | N/A | N/A | 0.3 |
| **E2-17G** | 27 | 21 | 48 | 42 | 14 | 21 | 0.23 | 0.74 | 1.7 | 0.52 | 0.06 | 2.24 | 0.005 | 3.1 |
| **E2-3S** | 8.9 | 14 | 5.0 | 13 | 3.4 | 5.3 | 0.23 | 0.74 | 1.1 | 0.22 | 0.03 | N/A | N/A | 0.2 |
| **E1-3S** | 25 | 63 | 74 | 76 | 4.6 | 27 | 0.36 | 0.16 | 0.2 | 0.04 | 0.005 | 0.53 | 0.0001 | 0.1 |
| **E2-17S** | 6.9 | 17 | 8.2 | 28 | 4.7 | 3.3 | N/A | N/A | N/A | N/A | N/A | N/A | N/A | N/A |
| **E1** | 32 | 20 | 5.0 | 26 | 13 | 9.7 | 0.57 | 1.15 | 0.4 | 0.04 | 0.005 | 2.5 | 1 | 0.1 |
| **E2** | 19 | 14 | 9.7 | 14 | 6.1 | 9.5 | 1.22 | 2.27 | 0.65 | 0.16 | 0.02 | 2.5 | 1 | 0.3 |
| **EE2** | 31 | 46 | 49 | 62 | 7.2 | 25 | 1.51 | 7.55 | N/A | N/A | N/A | 3.22 | 2 | 0.2 |
| **E3** | 37 | 59 | 26 | 52 | 3.6 | 10 | 0.41 | 1.13 | 0.85 | 0..24 | 0.03 | 5.04 | 1 | 1.5 |

(a)LOD - Limit of detection, determined using the most abundant product ion.

(b) Sample volume.

(c) DW - Drinking water; (d) RW - River water; (e) WW - Wastewater; (f) Eff - Effluent; (g) GW - Groundwater.

N/A - Not analyzed

N/D - No Information

* IDL - Instrument detection limit

A – Kuster et al.[[43](#_ENREF_43)];

B – Gentili et al.[[33](#_ENREF_33)];

C – Mozaz et al.[[42](#_ENREF_42)];

D – Díaz-Cruz et al. [[2](#_ENREF_2)];

E – Isobe et al.[[40](#_ENREF_40)];
