# Supplementary material for: Analysis of steroid hormones and their conjugated forms in water and urine by on-line solid-phase extraction coupled to liquid chromatography tandem mass spectrometry
Source: Chem Cent J. 2016 May 6;10:30. doi: 10.1186/s13065-016-0174-z (PMC4859969; doi:10.1186/s13065-016-0174-z)
Supplement: Supplementary file 7 — 10.1186/s13065-016-0174-z Comparison of measured quantification limits (LOQs) of the studied estrogens with other methods found in the literature for water samples. Concentrations in ng L−1. [file 13065_2016_174_MOESM7_ESM.docx]

Table 5 – Comparison of measured quantification limits (LOQs) of the studied estrogens with other methods found in the literature for water samples. Concentrations in ng L^-1^.

| **Estrogens** | **LOQ ^(a)^** |  |  |  |  |  |  |  |  |  |  |  |
| --- | --- | --- | --- | --- | --- | --- | --- | --- | --- | --- | --- | --- |
|  | Present Method | | | | | | F | | | G | | |
|  | HPLC | DW ^(c)^ | RW ^(d)^ | WW ^(e)^ | HPLC | RW ^(d)^ | WW ^(e)^ | WW^(e)^ | Eff^(f)^ | WW^(e)^ | Eff^(f)^ | RW ^(g)^ |
|  | 1 mL^(b)^ | 1 mL^(b)^ | 1 mL^(b)^ | 1 mL^(b)^ | 5 mL^(b)^ | 5 mL^(b)^ | 50 mL ^(b)^ | 100 mL^(b)^ | 250 mL ^(b)^ | 150 mL^(b)^ | 400 mL^(b)^ | 4000 mL^(b)^ |
| **E3-3S** | 21 | 39 | 21 | 123 | 28 | 19 | 5 | 3 | 1 | N/A | N/A | N/A |
| **E2-17G** | 81 | 63 | 144 | 126 | 42 | 63 | 6 | 3 | 1 | N/A | N/A | N/A |
| **E2-3S** | 27 | 42 | 15 | 39 | 10 | 16 | 3 | 2 | 1 | N/A | N/A | N/A |
| **E1-3S** | 75 | 189 | 222 | 228 | 14 | 81 | 2 | 0.8 | 0.3 | N/A | N/A | N/A |
| **E2-17S** | 21 | 51 | 25 | 84 | 14 | 9.9 | N/A | N/A | N/A | N/A | N/A | N/A |
| **E1** | 96 | 60 | 15 | 78 | 39 | 29 | 2 | 1 | 0.5 | 0.2 | 0.08 | 0.008 |
| **E2** | 57 | 42 | 29 | 42 | 18 | 29 | 4 | 2 | 1 | 0.6 | 0.2 | 0.02 |
| **EE2** | 93 | 138 | 147 | 186 | 22 | 75 | N/A | N/A | N/A | 0.9 | 0.3 | 0.03 |
| **E3** | 111 | 177 | 78 | 156 | 11 | 30 | 4 | 2 | 1 | 0.6 | 0.2 | 0.02 |

(a)LOQ - Limit of Quantification, determined using the most abundant product ion.

(b) Sample volume.

(c) DW - Drinking water; (d) RW - River water; (e) WW - Wastewater; (f) Eff - Effluent; (g) GW - Groundwater.

N/A - Not analyzed

F - D’Asenzo et al. [[52](#_ENREF_52)];

G - Baronti et al.[[15](#_ENREF_15)];
