# Supplementary material for: Analysis of steroid hormones and their conjugated forms in water and urine by on-line solid-phase extraction coupled to liquid chromatography tandem mass spectrometry
Source: Chem Cent J. 2016 May 6;10:30. doi: 10.1186/s13065-016-0174-z (PMC4859969; doi:10.1186/s13065-016-0174-z)
Supplement: Supplementary file 8 — 10.1186/s13065-016-0174-z Accuracy for the selected estrogens for all waters tested. [file 13065_2016_174_MOESM8_ESM.docx]

Table 6 – Accuracy for the selected estrogens for all waters tested.

**Accuracy ^(a)^**

| Estrogens | DW ^(b)^ | RW ^(c)^ | WW ^(d)^ | WW* ^(d)^ | RW** ^(c)^ |
| --- | --- | --- | --- | --- | --- |
|  | 1 mL^(e)^ | 1 mL^(e)^ | 1 mL^(e)^ | 1 mL^(e)^ | 5 mL^(e)^ |
| E3-3S | 18 | -2.3 | 70 | 30 | -4.5 |
| E2-17G | 7.9 | -5.9 | -3.9 | -11 | 4.1 |
| E2-17S | 7.1 | -1.6 | 8.0 | 0.4 | -6.2 |
| E1-3S | 1.1 | 2.4 | X | -0.1 | -4.5 |
| E2-3S | 4.5 | -2.0 | 3.9 | -2.5 | -9.4 |
| E3 | 7.3 | 0.8 | -75 | -36 | -6.2 |
| E2 | 4.5 | 2.7 | 0.7 | 4.3 | -11 |
| E1 | 0.5 | 0.6 | -15 | -5.5 | -6.9 |
| EE2 | 7.2 | 11 | 2 | 0.2 | 1.2 |

(a) Accuracy for the selected estrogens (C = 200 ng L^-1^, n = 10). * (C = 1000 ng L^-1^, n = 10). ** (C = 50 ng L^-1^, n = 10).

(b) DW - drinking water; (c) RW - river water; (d) WW – wastewater (e) Sample volume.
