# Supplementary material for: Analysis of steroid hormones and their conjugated forms in water and urine by on-line solid-phase extraction coupled to liquid chromatography tandem mass spectrometry
Source: Chem Cent J. 2016 May 6;10:30. doi: 10.1186/s13065-016-0174-z (PMC4859969; doi:10.1186/s13065-016-0174-z)
Supplement: Supplementary file 9 — 10.1186/s13065-016-0174-z Matrix Effects for the selected estrogens for all waters tested (in percentage). [file 13065_2016_174_MOESM9_ESM.docx]

Table 7 – Matrix Effects for the selected estrogens for all waters tested (in percentage).

| **Estrogens^(a)^** | **DW ^(b)^** | **RW ^(c)^** | **RW* ^(c)^** | **WW ^(d)^** |
| --- | --- | --- | --- | --- |
|  | **1 mL^(e)^** | **1 mL^(e)^** | **5 mL^(e)^** | **1 mL^(e)^** |
| E3-3S | 111 | 85 | 84 | 153 |
| E2-17G | 90 | 91 | 107 | 87 |
| E2-17S | 100 | 93 | 97 | 98 |
| E1-3S | 99 | 100 | 154 | 148 |
| E2-3S | 128 | 120 | 95 | 78 |
| E3 | 113 | 107 | 91 | 120 |
| E2 | 117 | 113 | 99 | 103 |
| E1 | 95 | 99 | 100 | 78 |
| EE2 | 137 | 141 | 86 | 94 |

(a) Matrix effects for the selected estrogens (C = 200 ng L^-1^, n = 10). * (C = 50 ng L^-1^, n = 10).

(b) DW - drinking water; (c) RW - river water; (d) WW – wastewater (e) Sample volume.
