# Supplementary material for: Analysis of steroid hormones and their conjugated forms in water and urine by on-line solid-phase extraction coupled to liquid chromatography tandem mass spectrometry
Source: Chem Cent J. 2016 May 6;10:30. doi: 10.1186/s13065-016-0174-z (PMC4859969; doi:10.1186/s13065-016-0174-z)
Supplement: Supplementary file 10 — 10.1186/s13065-016-0174-z Calculated recovery values in percentage for the selected estrogens. BetaBasic column was used as SPE column for the on-line SPE–LC–MS/MS method. Recovery values were calculated comparing the same volume injection of those of urine samples diluted at least ten times. (n = 5). [file 13065_2016_174_MOESM10_ESM.docx]

Table 8 – Calculated recovery values in percentage for the selected estrogens. BetaBasic column was used as SPE column for the on-line SPE-LC-MS/MS method. Recovery values were calculated comparing the same volume injection of those of urine samples diluted at least ten times. (n = 5).

| Estrogens | Recovery (%) | | |
| --- | --- | --- | --- |
|  | 500 ng L^-1^ | 1000 ng L^-1^ | 5000 ng L^-1^ |
| E3-3S | 85 | 83 | NC |
| E2-17G | 94 | 90 | NC |
| E2-17S | 81 | 96 | 63 |
| E1-3S | 87 | 73 | NC |
| E2-3S | 92 | 93 | 64 |
| E3 | 114 | 109 | 115 |
| E2 | 117 | 99 | 97 |
| E1 | 110 | 94 | 94 |
| EE2 | 118 | 99 | 97 |

NC – not calculated because the signal was too strong
